# The Wavelet-Based Cluster Analysis for Temporal Gene Expression Data

**DOI:** 10.1155/2007/39382

**Published:** 2007-05-15

**Authors:** JZ Song, KM Duan, T Ware, M Surette

**Affiliations:** 1 Department of Animal and Avian Science, 2413 Animal Science Center, University of Maryland, College Park, MD 20742, USA; 2 Department of Microbiology and Infectious Diseases, and Department of Biochemistry and Molecular Biology, Health Sciences Centre, University of Calgary, Calgary, AB T2N 4N1, Canada; 3 Department of Mathematics, University of Calgary, Calgary, AB T2N 4N1, Canada

## Abstract

A variety of high-throughput methods have made it possible to generate detailed temporal expression data for a single gene or large numbers of genes. Common methods for analysis of these large data sets can be problematic. One challenge is the comparison of temporal expression data obtained from different growth conditions where the patterns of expression may be shifted in time. We propose the use of wavelet analysis to transform the data obtained under different growth conditions to permit comparison of expression patterns from experiments that have time shifts or delays. We demonstrate this approach using detailed temporal data for a single bacterial gene obtained under 72 different growth conditions. This general strategy can be applied in the analysis of data sets of thousands of genes under different conditions.

## 

[1,2,3,4,5,6,7,8,9,10,11,12,13,14,15,16,17,18,19,20,21,22,23,24,25,26,27,28,29]
